# Valosin-containing protein (VCP) promotes the growth, invasion, and metastasis of colorectal cancer through activation of STAT3 signaling

**DOI:** 10.1007/s11010-016-2746-6

**Published:** 2016-06-25

**Authors:** Qianfeng Fu, Yuling Jiang, Daxin Zhang, Xiuli Liu, Junfeng Guo, Jinlong Zhao

**Affiliations:** Department of Oncology, The First Affiliated Hospital of Harbin Medical University, No. 23 Youzheng Street, Harbin, 150001 Heilongjiang China; Department of Clinical Laborotary, The First Affiliated Hospital of Harbin Medical University, No. 23 Youzheng Street, Harbin, 150001 Heilongjiang China

**Keywords:** Valosin-containing protein, Colorectal cancer, Metastasis, Proliferation, STAT3

## Abstract

Valosin-containing protein (VCP) was previously shown to exhibit high expression in colorectal cancer (CRC) tissues as compared with that in normal tissues; however, the role of VCP in human CRC cells has remained to be elucidated. Two colorectal cancer cell lines HCT116 and RKO were used in the experiment. We introduced lentiviral constructs expressing VCP to infect RKO cells and lenti-shRNA targeting VCP into HCT116 cells, respectively. Cell proliferation, invasion, apoptosis, and cell cycle arrest were subsequently examined by MTT assay, transwell chamber assay, flow cytometry, and western blot analysis, respectively. Furthermore, a subcutaneous tumor mouse model and lung metastasis model was used to investigate the effects of VCP on the growth and metastasis of CRC cells in vivo. VCP knockdown was shown to inhibit cell proliferation, chemoresistance and invasion, and induce apoptosis in the HCT116 CRC cells, whereas VCP over-expression suppressed apoptosis and chemoresponse, promoted proliferation and invasion of the RKO CRC cells. In addition, in the subcutaneous tumor and lung metastasis mouse model, VCP knockdown in HCT116 cells suppressed carcinogenesis and metastasis in vivo. The findings of the present study indicated that VCP is very important for the proliferation and metastasis of CRC; therefore, targeting VCP and its downstream targets may represent novel therapies for the treatment of CRC.

**Electronic supplementary material:**

The online version of this article (doi:10.1007/s11010-016-2746-6) contains supplementary material, which is available to authorized users.

## Introduction

Colorectal cancer (CRC) is the third most common cancer and is tightly linked to lifestyle. Indeed, high-income countries such as the USA, New Zealand, Japan, and Western Europe show an incidence for CRC 10-fold higher than low-income and middle-income countries (Africa, South America, and South Asia) [1]. The disease typically develops over many years via a sequence of genetic changes, which is known as the adenoma–carcinoma sequence [2]. Every year, more than 1 million new cases are diagnosed. Although much effort has centered on probing the pathogenesis of the disease, the molecular mechanisms underlying the process are still largely unknown [3].

Valosin-containing protein (VCP) belongs to the AAA family (ATPase with multiple cellular activities) [4]. It is one of the most abundant proteins in eukaryotic cells and can interact with more than 30 different cellular proteins with various functions, including morphology alteration of nuclear and golgi membranes, transcriptional regulation, membrane fusion, programed cell death, ubiqutin/proteasome-dependent protein degradation, and ER-associated degradation [5–8]. In addition, VCP is associated with the various cellular pathologies and disease states including neurodegenerative disorders [9, 10], pulmonary conditions [11], and other protein misfolding disorders [12, 13]. Additionally, clinical studies have identified a correlation between elevated VCP expression and the progression, prognosis, and metastatic potential of esophageal carcinoma [14], colorectal carcinoma [15], and prostate cancer [16]. Although Yamamoto S et al. demonstrated that VCP is upregulated and high VCP expression levels are associated with poor prognosis of CRC patients [15], the detailed function of VCP in human CRC remains unclear.

Signal transducers and activators of transcription (STATs) are cytoplasmic transcription factors, and STATs are key mediators of cytokine and growth factor signaling pathways [17]. STATs are latent transcription factors activated by phosphorylation of a conserved tyrosine residue. Experimental and clinical data have revealed the oncogenic potential of STAT3 through over-expression and constitutive activation in a variety of human malignancies [18]. When phosphorylated at tyrosine^705^, STAT3 undergoes translocation from the cytosol to the nucleus, where it functions as a pivotal transcription factor upregulating gene transcription [19]. Increasing evidences suggest that STAT3 signaling is aberrant in human CRC cells and CRC tissues with prolonged and sustained STAT-3 phosphorylation [20, 21]. So, STAT3 is a promising candidate for CRC targeted therapy.

In the present study, we demonstrate that VCP promotes proliferation and metastasis in CRC, and VCP knockdown induces G1 phase arrest and apoptosis in CRC through STAT3 dephosphorylation, both in vitro and in vivo.

## Materials and methods

### Cell lines and regents

HCT116 and RKO CRC cell lines were obtained from Shanghai Bioleaf Biotech Co., Ltd (Shanghai, China). HCT116 and RKO were p53 wild-type cells. HCT116 was obtained from a male colonic carcinoma patient [22]. RKO was obtained from a poorly differentiated colon carcinoma patient [23]. CRC cell lines were incubated in DMEM (Gibco, Invitrogen Company, Grand Island, NY) containing 10 % heat-inactivated fetal bovine serum supplemented with 1 % penicillin–streptomycin solution (Gibco) at 37 °C in a humidified atmosphere of 5 % CO_2_. 5-FU was purchased from Sigma-Aldrich (St. Louis, MO).

### Lentiviral infection

Human lenti-VCP, lenti-shVCP, lenti-vector control, and lenti-shNC were designed and purchased from GeneChem Technologies (Shanghai, China). The transfection was performed according to standard procedures. Following lentiviral infection, single-cell clonal isolates were selected in the presence of puromycin for 2–4 weeks.

### MTT assay

CRC cells were seeded in 96-well plates at a density of 2 × 10^3^ cells/well. After 24, 48, and 72 h, the medium was replaced with 200 μL of 10 % FBS supplemented DMEM containing 0.5 mg/mL MTT (Sigma-Aldrich), and cells were incubated in the CO_2_ incubator at 37 °C for 4 h. Medium was removed, the reduced MTT was solubilized in 100 μL per well of DMSO (Sigma-Aldrich), and measured absorbance at 570 nm by a IMARK microplate reader (Bio-Rad, Hercules, CA, USA).

### Western blot

For preparing total cell lysates, cells were lysed in lysis buffer (Invitrogen), incubated on ice for 30 min and centrifuged for 20 min to remove cell debris. Total cell lysate was subjected to SDS-polyacrylamide gel electrophoresis. The proteins were then electro-transferred to polyvinylidene difluoride membrane (Millipore, Billerica, MA) and incubated overnight with antibodies at 4 °C. Subsequently, the membranes were incubated with secondary antibodies for 1 h at room temperature and the signal was detected using an enhanced chemiluminescence detection kit (Pierce, Rockford, IL). The primary antibodies: VCP, Ki-67, E-cadherin, N-cadherin, and vimentin were purchased from Abcam (Cambridge, MA). Cyclin D1, CDK4, cyclin E, CDK2, PCNA, Bcl-2, Bax, Bcl-xL, and actin were purchased from Santa Cruz Biotechnology (Dallas, Texas). STAT3, p-STAT3 (Tyr705), p21, cleaved-caspase-3, and cleaved-PARP were purchased from Cell Signaling Technology (Danvers, MA). The secondary antibodies, antimouse IgG-HRP, and antirabbit IgG-HRP were purchased from Santa Cruz Biotechnology.

### Cell cycle and apoptosis analysis

Cell cycle and apoptosis assays were performed as described elsewhere [24]. Annexin V–FITC apoptosis kit and cell cycle analysis kit were purchased from Becton–Dickinson, San Diego, CA, USA. Cells were analyzed on a Becton–Dickinson FACS Caliber flow cytometer (BD Biosciences).

### Immunohistochemistry analysis

Immunohistochemistry was performed using Ki-67, cleaved-caspase-3, and CD31 antibodies. In brief, tissue sections were deparaffinized in xylene and rehydrated with ethanol. Tissue sections were then preincubated with 10 % normal goat serum in PBS (pH 7.5) followed with incubation with primary antibody overnight at 4 °C. Tissue sections were then stained with biotinylated secondary antibody (Vector lab, Burlingame, CA) for 1 h at room temperature, followed by the Vectastain Elite ABC reagent (Vector lab) for 30 min. The peroxidase reaction was developed with diaminobenzidine (DAB kit; Vector lab) and the slides were counterstained with hematoxylin (Sigma).

### Cell invasion assay

Invasion was measured using 24-well BioCoat cell culture inserts (BD Biosciences) with an 8-μm-porosity polyethylene terephthalate membrane coated with Matrigel Basement Membrane Matrix (BD Biosciences). The invasion assay was performed as previously described [25].

### Immunofluorescence

Briefly, cells seeded on coverslips were fixed with 4 % (w/v) paraformaldehyde (Sigma) for 10 min and permeabilized with 0.1 % (v/v) Triton X-100 for 5 min at room temperature. The cells were then incubated overnight with primary antibodies at 4 °C, followed by incubation with fluorescent secondary antibody (invitrogen) for 1 h at room temperature. After final washes with PBS, the coverslips were mounted using an antifade mounting solution containing 4′,6-diamidino-2-phenylindole (DAPI; Vector lab) and images were examined and captured.

### Subcutaneous CRC experiments

The study was approved by the ethics committee of The First Affiliated Hospital of Harbin Medical University (Harbin, China). The average weight 20 g, 6-week-old female athymic nude mice (BALBc nu/nu; Experimental Animal Laboratories, Shanghai, China) were used in all experiments (8 mice for each group). The animals were maintained in a specific pathogenfree environment. The mice were fed with autoclaved food and water. The animal room was kept at 20–22 °C under a 12-hour light/dark cycle. Each mouse was injected subcutaneously with stably transfected CRC cells and control cells (4 × 10^6^). The volume of tumors were monitored and measured by vernier caliper, and tumor volume (mm^3^) was calculated using the standard formula: length × width × height × 0.5236. All mice sacrificed by CO_2_ six weeks after implantation, snap-frozen and paraffin-embedded tumor tissue blocks were obtained for further analysis.

### In vivo invasive assay

To evaluate long-distance lung metastasis, CRC cells (1 × 10^6^/0.2 mL) were injected into nude mice (*n* = 8/group) by way of tail vein to imitate lung metastasis. Mice were sacrificed at 8 weeks, and the lung metastases were confirmed by H&E staining.

### Statistical analysis

Statistical analysis was performed with the GraphPad Prism software package (v. 4.02; San Diego, CA, USA) or SPSS 16.0 software (Chicago, IL, USA). Values are expressed as the mean ± SD. Comparisons between multiple groups were made using a one-way analysis of variance, followed by Dunnet’s *t* test. *P* < 0.05 was considered to indicate a statistically significant difference between values.

## Results

### VCP promotes cell proliferation and VCP knockdown induces G1 phase arrest in CRC cell lines

**Fig. 1:**
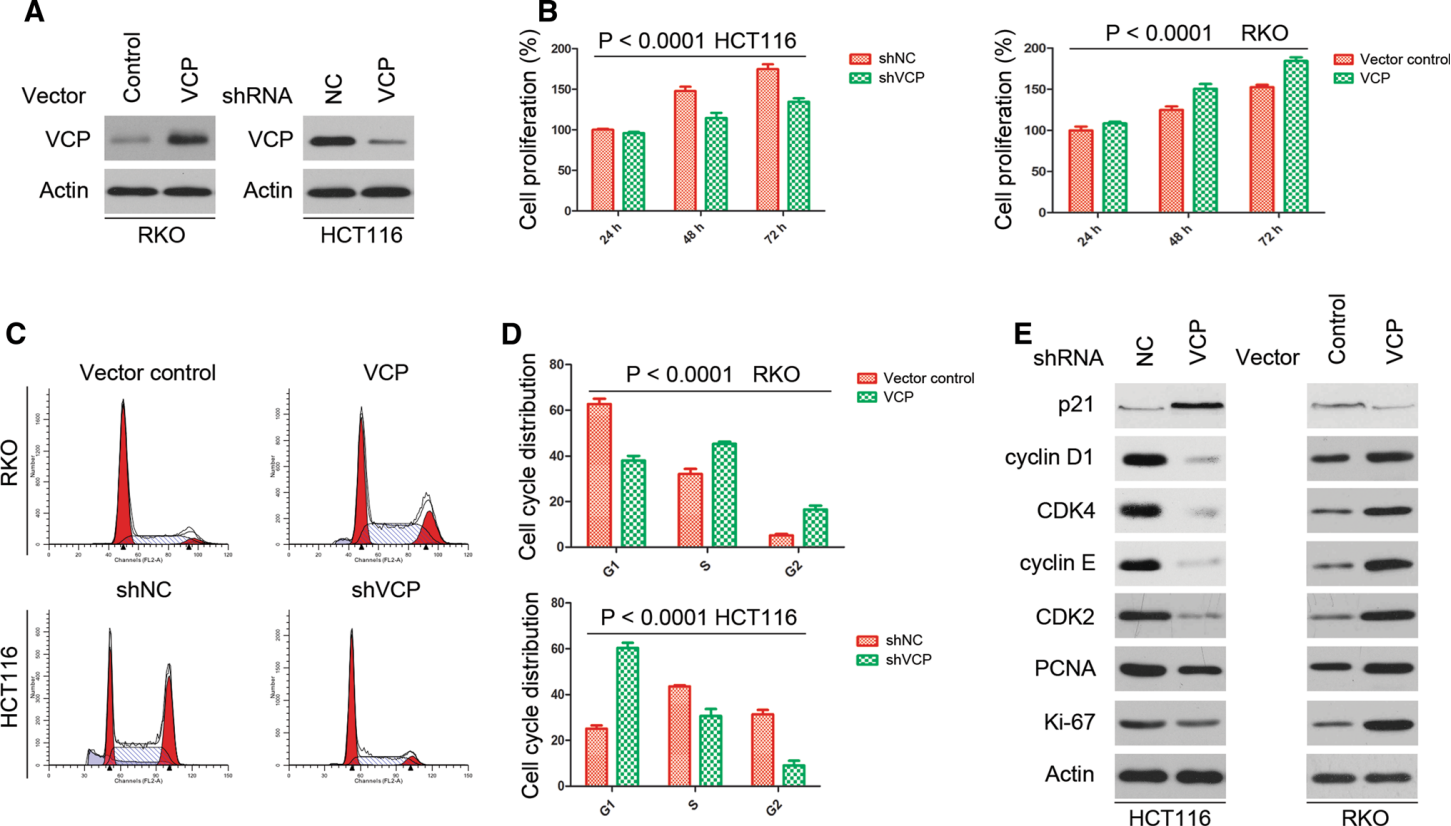
Effects of VCP on cell proliferation, cell cycle-associated protein expression, and cell cycle progression in HCT116 and RKO cells. **a** The expression level of VCP was detected by western blot after lenti-VCP or shVCP transfection. Actin was used as the internal control. Western blot was repeated three times. **b** Effects of VCP knockdown and over-expression on the proliferation of CRC cells. Cell proliferation after transfection was assessed by MTT assay. Values are expressed as the mean ± SD of three independent experiments. **c** and **d** Representative images of the cell cycle analysis of indicated CRC cells. VCP knockdown induced G1 cell cycle arrest in HCT116 cells, and VCP over-expression increased in the number of RKO cells in S phase. The percentage of cells in each phase of the cell cycle is presented as the mean ± SD from three independent experiments. **e** CRC cells were subjected to western blot analysis for the indicated protein expression. Actin was used as the internal control. Western blot was repeated three times

As shown in Fig. 1a and Supplementary Fig. 1a, the basal level of VCP is higher in HCT116 than in RKO cells. So, we chose these two cell lines with different VCP basal levels. To determine whether VCP expression had an effect on CRC cell progression, we generated lentiviral constructs expressing VCP to infect RKO cells, and we introduced lenti-shRNA targeting VCP into HCT116 cells. The levels of VCP after transfection were determined by western blot (Fig. 1a).

To determine whether there was an association between VCP expression and CRC cell proliferation, an MTT assay was performed. RKO cells transfected with lenti-VCP exhibited a marked increase in cell proliferation as compared with that in the control group (Fig. 1b). In addition, there was a significant decrease in cell proliferation in the HCT116 cells transfected with shVCP (Fig. 1b). Flow cytometry revealed an increase in the number of lenti-VCP-transfected RKO cells in S phase, as compared with that in the control group (Fig. 1c, d). Conversely, there was an increase in the number of shVCP-transfected HCT116 cells in G1 phase (Fig. 1c, d). Concurrently, the expression levels of cell cycle arrest-associated proteins were examined by western blot analysis (Fig. 1e and Supplementary Fig. 1b). The expression of p21 increased in shVCP-transfected HCT116 cells, and p21 level decreased in lenti-VCP-transfected RKO cells. In addition, the protein expression levels of cyclin D1, cyclin E, CDK2, CDK4, Ki-67, and PCNA were higher in lenti-VCP-transfected RKO cells, as compared with those in control cells. Conversely, the expression levels were lower in shVCP-transfected HCT116 cells as compared with those in control cells, which was concordant with the results from the MTT and cell cycle assays. These results suggested that VCP may promote cell proliferation and VCP knockdown induce G1 cell cycle arrest in CRC cells.

### VCP knockdown increases the percentage of apoptotic CRC cells, and promotes chemoresponse to 5-FU in CRC cells

**Fig. 2:**
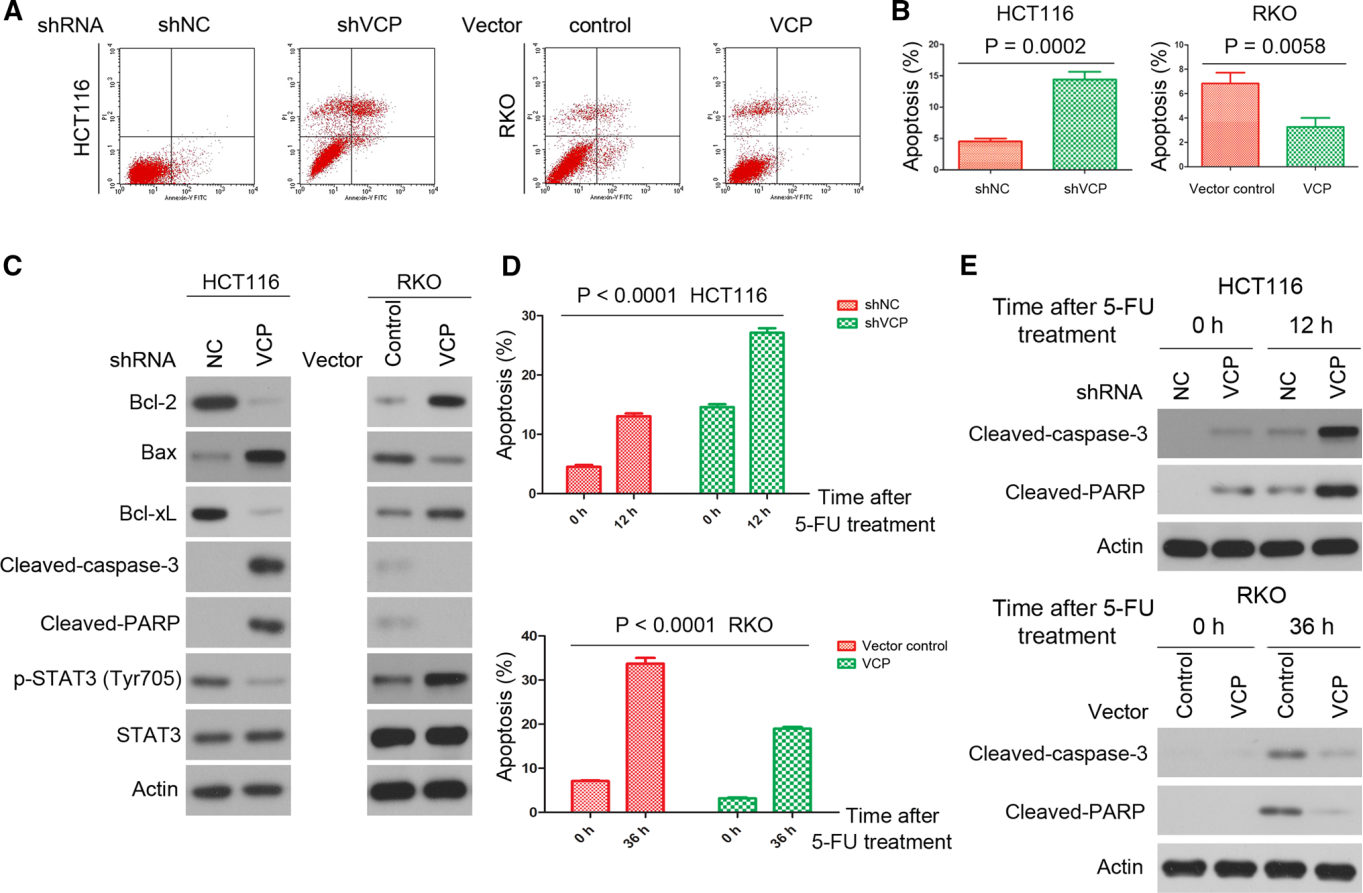
VCP knockdown induces apoptosis in CRC cells and promotes 5-FU-induced apoptosis in CRC cells. **a** and **b** Flow cytometry results of annexin V-PI staining of CRC cells following transfection with lenti-VCP or shVCP. An increase in the percentage of apoptotic cells following transfection with shVCP is shown. Values are expressed as the mean ± SD of three independent experiments. **c** Changes in protein expression levels of anti- or proapoptotic proteins following transfection with lenti-VCP or shVCP. Actin was used as the internal control. Western blot was repeated three times. **d** The percentage of apoptotic cells was determined by annexin V-PI staining after transfection and 5-FU (500 μM) treatment. Values are expressed as the mean ± SD of three independent experiments. **e** Indicated protein expression was detected by western blot after transfection and 5-FU (500 μM) treatment. Actin was used as the internal control. Western blot was repeated three times

The number of apoptotic CRC cells was measured using an Annexin V/PI Apoptosis Detection kit and flow cytometry at three days posttransfection. shVCP-transfected HCT116 cells exhibited a relatively high rate of cell apoptosis as compared with control cells (Fig. 2a, b). Conversely, RKO cells transfected with lenti-VCP exhibited a relatively low rate of apoptosis as compared with that of the control cells (Fig. 2a, b).

In addition, western blot analysis was performed to determine the expression levels of the following apoptosis-associated proteins in CRC cells: Bcl-2, Bax, Bcl-xL, cleaved-PARP, cleaved-caspase-3, STAT3, and p-STAT3. At three days posttransfection, the expression levels of Bcl-2 and Bcl-xL were significantly decreased in the shVCP-transfected HCT116 cells as compared with control cells, whereas the expression levels of Bax, cleaved-PARP, and cleaved-caspase-3 were increased. Furthermore, shVCP-transfected HCT116 cells exhibited lower expression levels of p-STAT3, whereas the expression levels of total STAT3 were unaffected (Fig. 2c and Supplementary Fig. 1c). Conversely, three days posttransfection, Bcl-2, Bcl-xL, and p-STAT3 expression were upregulated, and the expression levels of Bax, cleaved-PARP, and cleaved-caspase-3 were decreased in the lenti-VCP-transfected RKO cells (Fig. 2c and Supplementary Fig. 1c). These results indicated that the VCP knockdown-dependent increase in the rate of CRC cell apoptosis may be partially mediated by regulation of Bcl-2, Bcl-xL, Bax, cleaved-PARP, cleaved-caspase-3, and p-STAT3.

To investigate the potential role of VCP in CRCs, the effect of VCP on the cellular response toward chemotherapeutic agents was determined in HCT116 and RKO cells. Notably, lenti-VCP transfection markedly reduced 5-FU-induced apoptosis in RKO cells, and VCP knockdown markedly increased 5-FU-induced apoptosis in HCT116 cells (Fig. 2d). The effect of VCP on 5-FU-mediated apoptosis was further clearly demonstrated by the detection of cleaved-PARP and cleaved-caspase-3 in CRC cells transfected with lenti-VCP or shVCP (Fig. 2e and Supplementary Fig. 1d).

### VCP promotes CRC growth in vivo

**Fig. 3:**
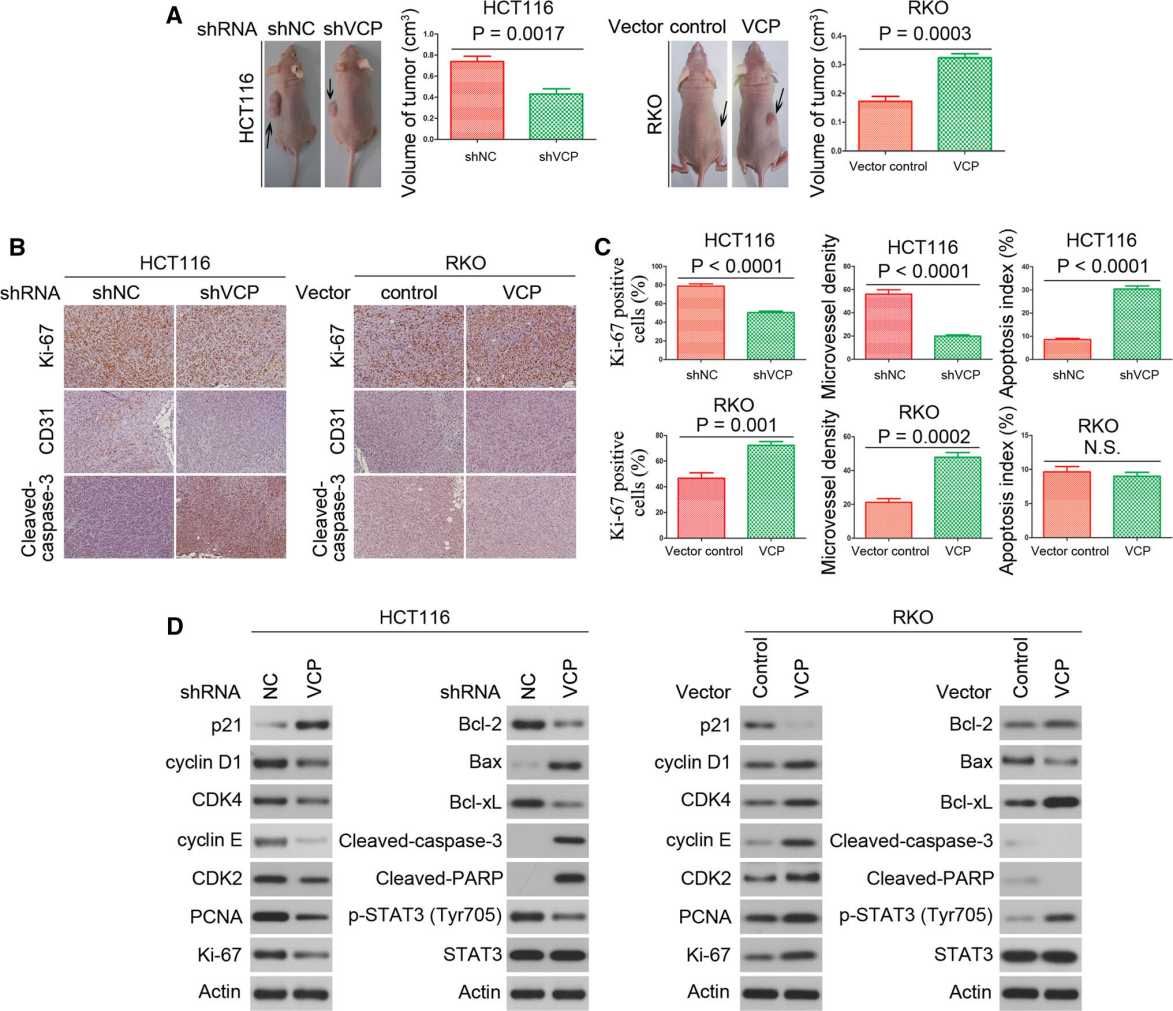
VCP promotes proliferation of CRC in vivo. **a** Representative images of xenograft tumors were shown for the indicated CRC cells. The histogram showed the size of tumors for the indicated CRC cells. Values are expressed as the mean ± SD of experiments performed in triplicate. **b** Tumors from the different groups were immunostained for cleaved-caspase-3, CD31 and Ki-67. Images are representative of three independent experiments. **c** Quantification of immunostaining in (**b**). CD31-stained microvessels were counted to record microvessel density, apoptotic cells were counted to give the apoptosis index and cells expressing Ki-67 were counted to calculate the Ki-67 positive cells. **d** Western blot was performed to detect the protein expression levels of the indicated molecules from tumor samples. Actin was used as the internal control. Western blot was repeated three times

The present study further examined the effects of VCP on CRC growth by establishing HCT116 and RKO xenograft nude mouse CRC models. Mice injected with HCT116 cells which were transfected with shVCP exhibited significantly smaller tumors as compared with control group (Fig. 3a). Conversely, Mice injected with RKO cells over-expressing VCP exhibited significantly larger tumors as compared with control group (Fig. 3a). Immunohistochemical analysis was used to stain Ki-67 to determine cell proliferation; cleaved-caspase-3 to detect apoptotic cells; and CD31 to detect tumor microvessels. There were fewer Ki-67 positive cells, more apoptotic cells, and fewer CD31-stained vessels in tumors with VCP knockdown, as compared with the control tumors (Fig. 3b, c). In tumors over-expressing VCP, there were more Ki-67 positive cells and CD31-stained vessels as compared with the control tumors (Fig. 3b, c). In addition, western blot showed the levels of p-STAT3, cyclin D1, cyclin E, CDK2, CDK4, PCNA, Ki-67, Bcl-2, and Bcl-xL were decreased in the VCP knockdown tumors, and increased in the VCP over-expressing tumors (Fig. 3d and Supplementary Fig. 1e). In addition, the expression levels of p21, Bax, cleaved-PARP, and cleaved-caspase-3 were increased in the VCP knockdown tumors, and decreased in the VCP over-expressing tumors (Fig. 3d and Supplementary Fig. 1e). These results indicate the functional significance of VCP, and its high propensity to promote proliferation in CRC.

### VCP knockdown inhibits CRC metastasis in vitro and in vivo

**Fig. 4:**
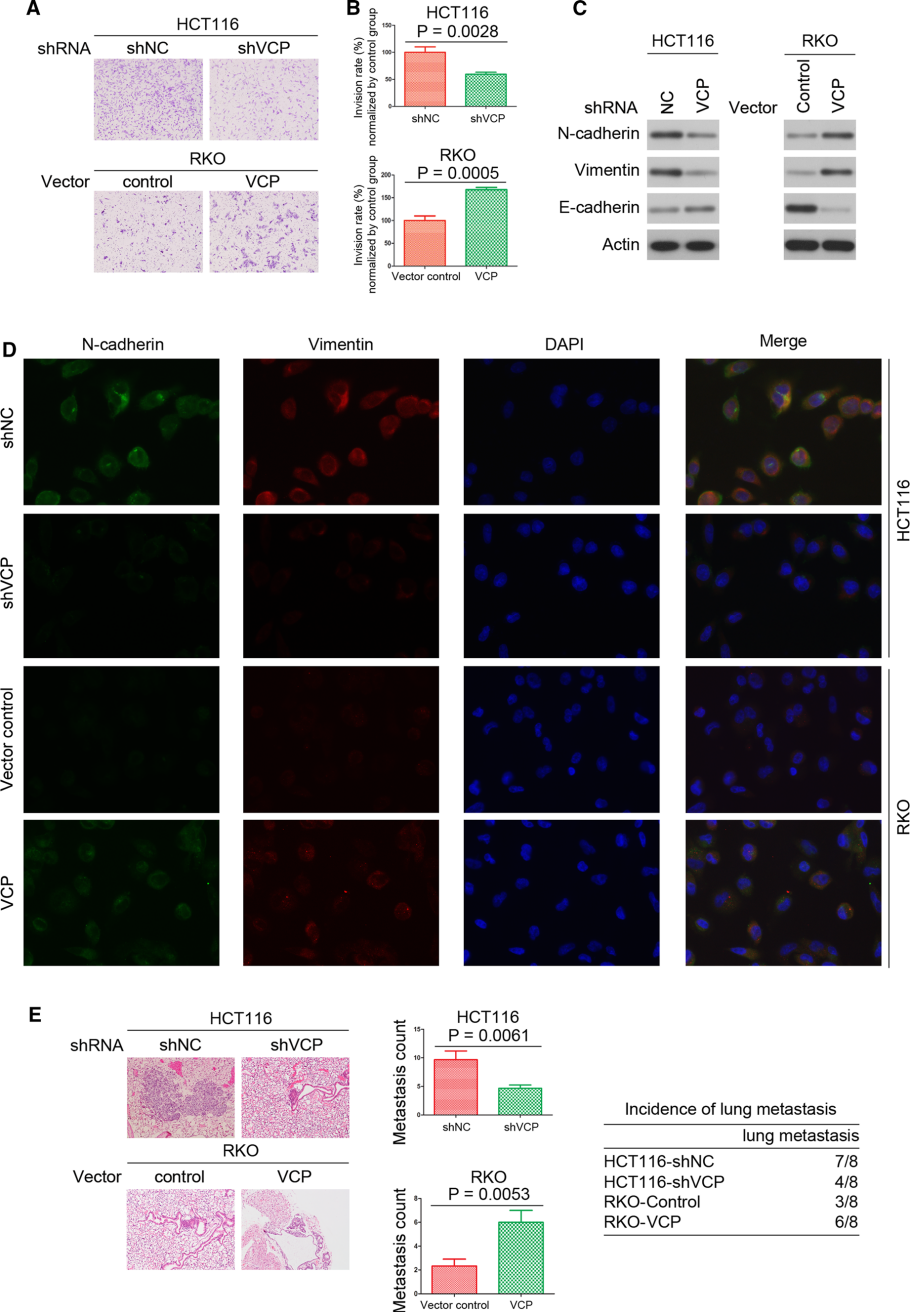
VCP promotes metastasis of CRC in vitro and in vivo. **a** and **b** Cell invasion experiments demonstrated that transfection with shVCP significantly inhibited the invasive capacity of HCT116 cells, and transfection with lenti-VCP promoted the invasive capacity of RKO cells. Values are expressed as the mean ± SD of experiments performed in triplicate. **c** Protein expression levels of N-cadherin, vimentin, and E-cadherin in shVCP-transfected HCT116 cells and lenti-VCP transfected RKO cells. Actin was used as the internal control. Western blot was repeated three times. **d** Single and merged images were taken to show immunofluorescence staining of N-cadherin (*green*) and vimentin (*red*), accompanied by nuclear staining (*blue*) with DAPI. **e** Representative H&E images of lung metastases were shown for indicated cell lines. The histogram showed the number of metastatic nodules. The table showed incidence of lung metastasis in different groups. (Color figure online)

The association between VCP expression and CRC cell invasion was detected using a Matrigel invasion assay. The number of invaded shVCP-transfected HCT116 cells was significantly lower as compared with control group (Fig. 4a, b), thus suggesting that the percentage of invaded cells decreased in VCP knockdown cells. Furthermore, the invasiveness of lenti-VCP-transfected RKO cells was increased as compared with that of control cells (Fig. 4a, b).

The epithelial–mesenchymal transition (EMT) associated markers vimentin, N-cadherin, and E-cadherin have essential roles in the invasion of tumor cells. Therefore, the present study examined the expression levels of vimentin, N-cadherin, and E-cadherin in CRC cells by western blot. The protein expression levels of vimentin and N-cadherin were downregulated in shVCP-transfected HCT116 cells as compared with control group, and E-cadherin expression levels were upregulated (Fig. 4c and Supplementary Fig. 1f). In addition, in lenti-VCP-transfected RKO cells, the expression levels of vimentin and N-cadherin were significantly increased, and expression levels of E-cadherin were significantly decreased (Fig. 4c and Supplementary Fig. 1f). As shown by immunofluorescence (Fig. 4d), transfection with lenti-VCP markedly increased N-cadherin and vimentin expression in RKO cells, and transfection with shVCP significantly decreased N-cadherin and vimentin expression in HCT116 cells, which was in concordance with the results of the western blot analysis. These results suggested that VCP may promote CRC cell invasion in vitro.

Next, we tested whether VCP could promote metastasis in vivo. The tumor volume, number of nodules, and the incidence of lung metastasis were drastically decreased in VCP knockdown HCT116 cells. The tumor volume, number of nodules, and the incidence of lung metastasis were significantly increased in RKO cells which were transfected with lenti-VCP (Fig. 4e).

## Discussion

VCP plays a key role in the ubiquitin-dependent proteasome degradation pathway [8]. In particular, VCP inhibits apoptosis after stimulation with cytokines such as tumor necrosis factor via degradation of inhibitor кBα, an inhibitor of nuclear factor-кB (NF-кB). Murine osteosarcoma cells transfected with the *VCP* gene exhibited constant activation of NF-кB, rapid degradation of phosphorylated-inhibitor кBα, decreased apoptosis rates after tumor necrosis factor α stimulation, and increased metastatic potential [7]. VCP is overexpressed in many solid tumors, including prostate and pancreatic cancers [16, 26], esophageal carcinomas [14], and osteosarcoma [7]. Recent studies have also indicated that VCP expression may be an independent prognostic factor for overall survival in non-small cell lung carcinoma [27, 28]. Yamamoto S et al. reported that the level of VCP is associated with the prognosis of CRC [15]. However, the exact mechanisms underlying the effects of VCP on CRC are yet to be elucidated. Therefore, it is required to study the role of VCP in the regulation of CRC cell growth, survival, and invasion.

In order to test the function of VCP in CRC, shVCP was transfected into HCT116 cells. In the present study, MTT, flow cytometric and invasive assays demonstrated that downregulation of VCP resulted in the inhibition of cell proliferation, induction of apoptosis, and suppression of invasiveness. These results suggested that VCP has an important role in the regulation of tumorigenesis of CRC. Further evidence regarding this finding was obtained from RKO cells with VCP over-expression. Transfection of RKO cells with lenti-VCP was used to upregulate VCP expression, which resulted in increased cell proliferation and invasiveness, as well as decreased levels of apoptosis. VCP knockdown also markedly increased 5-FU-induced apoptosis in HCT116 cells, and over-expression of VCP promotes chemoresistance in cultured RKO cells. Therefore, VCP could be an important factor contributing to the chemoresistance in CRCs. Furthermore, in a subcutaneous mouse tumor model, VCP knockdown significantly reduced subcutaneous tumor growth, and VCP over-expression promoted subcutaneous tumor growth.

To provide further evidence regarding the mechanisms underlying the effects of VCP on CRC cells, the present study examined the expression levels of proteins associated with CRC cell proliferation (p21, cyclin D1, CDK4, cyclin E, CDK2, Ki-67, and PCNA) and apoptosis (Bcl-2/Bax, Bcl-xL, cleaved-PARP, cleaved-caspase-3, p-STAT3, and STAT3) by western blot analysis. The expression of p21 increased after shVCP transfection and decreased after lenti-VCP transfection. The protein expression levels of cyclin D1, CDK4, cyclin E, CDK2, and Ki-67, a biological tumor marker that indicates changes in tumor proliferation, were reduced in the shVCP-transfected HCT116 cells, and increased in the lenti-VCP transfected RKO cells. In addition, changes in the expression levels of PCNA, another well-known proliferation marker, were similar to those of Ki-67 in the shVCP-transfected HCT116 and the lenti-VCP transfected RKO cells.

The apoptosis-associated proteins, such as Bcl-2, Bax, Bcl-xL, cleaved-PARP, and cleaved-caspase-3 were also detected by western blot analysis. The results of the present study demonstrated that the upregulation of VCP induced an increase in the protein expression levels of Bcl-2, Bcl-xL, and also significantly decreased the expression levels of Bax, cleaved-PARP, and cleaved-caspase-3. Downregulation of VCP decreased the levels of Bcl-2, Bcl-xL, and increased the levels of Bax, cleaved-PARP, and cleaved-caspase-3. The STAT3 pathway is important in CRC, and numerous studies have been performed to demonstrate that the p-STAT3 expression levels were elevated in CRC in vitro and in vivo, and this expression was revealed to be correlated with the poor prognosis [20, 21, 29]. The present study revealed that VCP over-expression also induced STAT3 phosphorylation, whereas it did not affect the expression of total STAT3. Therefore, the increase in p-STAT3, together with the increased expression of Bcl-2, may explain why cells with high VCP expression are resistant to chemotherapy-induced apoptosis.

EMT, an absolute requirement for tumor invasion and metastasis, plays a key role in cancer progression [30, 31]. The present study demonstrated a positive correlation between vimentin, N-cadherin, and VCP expression, and a negative correlation between E-cadherin and VCP expression. These results indicated that the over-expression of VCP may promote the metastasis of CRC in vitro and in vivo through modulation of vimentin, N-cadherin, and E-cadherin.

In conclusion, the present study demonstrated that VCP may have an important role in the regulation of growth, chemoresistance, and metastasis of CRC. These findings indicated that VCP may serve as a potential therapeutic target in the treatment of CRC.

## Electronic supplementary material

Below is the link to the electronic supplementary material.

Supplementary material 1 (TIFF 30021 kb)
