# Supplementary figures and images for: Valosin-containing protein (VCP) promotes the growth, invasion, and metastasis of colorectal cancer through activation of STAT3 signaling
Source: Mol Cell Biochem. 2016 Jun 25;418:189–98. doi: 10.1007/s11010-016-2746-6 (PMC4927615; doi:10.1007/s11010-016-2746-6)

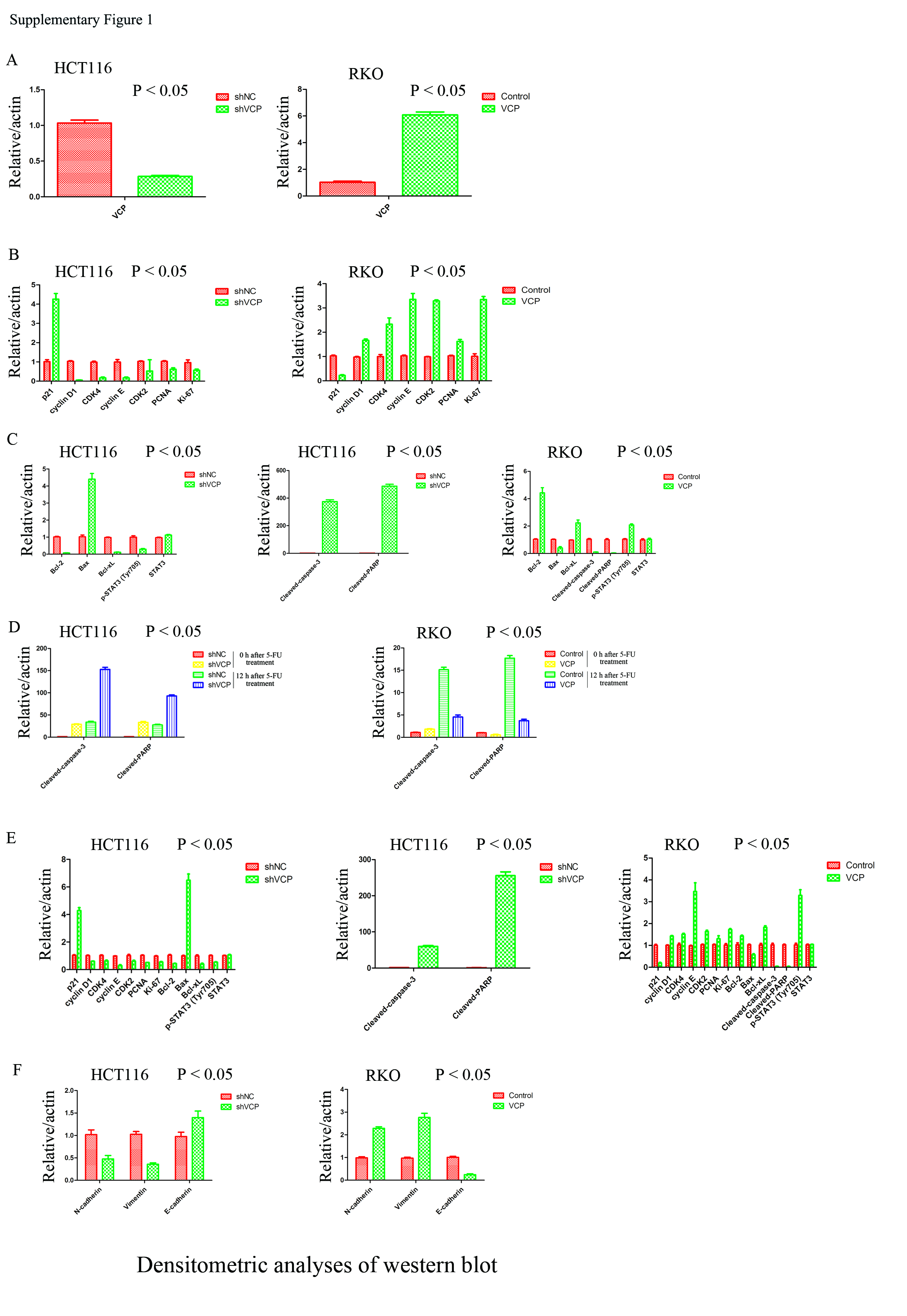

Supplement: Supplementary file 1 — Supplementary material 1 (TIFF 30021 kb) [file 11010_2016_2746_MOESM1_ESM.tif]
